# Reprocessable, Reworkable, and Mechanochromic Polyhexahydrotriazine Thermoset with Multiple Stimulus Responsiveness

**DOI:** 10.3390/polym12102375

**Published:** 2020-10-15

**Authors:** Li Chen, Siyao Zhu, Innocent Toendepi, Qiuran Jiang, Yi Wei, Yiping Qiu, Wanshuang Liu

**Affiliations:** 1 Shanghai Collaborative Innovation Center for High Performance Fiber Composites, Center for Civil Aviation Composites, Donghua University, 2999 North Renmin Road, Shanghai 201620, China; 2180072@mail.dhu.edu.cn (L.C.); 2180138@mail.dhu.edu.cn (S.Z.); muvinnot@icloud.com (I.T.); weiy@dhu.edu.cn (Y.W.); 2 Key Laboratory of Textile Science &Technology, Ministry of Education, College of Textiles, Donghua University, 2999 North Renmin Road, Shanghai 201620, China; jj@dhu.edu.cn (Q.J.); ypqiu@dhu.edu.cn (Y.Q.)

**Keywords:** polyhexahydrotriazine, vitrimers, recyclability, disulfide bonds

## Abstract

Developing recyclable, reworkable, and intelligent thermosetting polymers, as a long-standing challenge, is highly desirable for modern manufacturing industries. Herein, we report a polyhexahydrotriazine thermoset (PHT) prepared by a one-pot polycondensation between 4-aminophenyl disulfide and paraformaldehyde. The PHT has a glass transition temperature of 135 °C and good solvent resistance. The incorporation of dual stimuli-responsive groups (disulfide bond and hexahydrotriazine ring) endows the PHT with re-processability, re-workability, and damage monitoring function. The PHT can be repeatedly reprocessed by hot pressing, and a near 100% recovery of flexural strength is achieved. The PHT can also degrade in inorganic acid or organic thiol solutions at room temperature. The thermally reworkable test demonstrates that, after heating the PHT at 200 °C for 1 h, the residuals can be easily wiped off. Finally, the PHT exhibits a reversible mechanochromic behavior when damaged.

## 1. Introduction

Thermosetting polymers have widespread industrial applications such as structural adhesives, protective coatings, electronic packaging materials, and polymer matrices for advanced composites thanks to their remarkable mechanical and electrical performances as well as excellent heat, creep, and chemical resistance. However, conventional thermosetting polymers have permanent cross-linked structures consisting of irreversible covalent bonds, making them extremely difficult for recycling [1,2,3]. Currently, technological innovations greatly shorten the iterative circles of consumer products, especially for personal electronic equipment in which thermosetting polymers are largely used. Along with stricter environmental regulations, the intractability of thermosetting polymers has raised increasing concerns about disposal and recycling of discarded electronics and composite products. Besides recyclability, reworkable thermosetting polymers, which can be removed during the manufacturing process, are also desirable [2,4]. For example, it is a high cost to discard an assembled electronic component with only one faulty chip. The use of reworkable thermosetting polymers for electronic encapsulation will allow removal of an individual chip from the printed circuit board for replacement or repair. Therefore, both economic and environmental factors are driving the development of recyclable and/or reworkable thermosetting polymers.

Over the past two decades, incorporating dynamic covalent bonds has become an attractive avenue to endow thermosetting polymers with recyclability [5,6,7,8]. Such thermosetting polymers, which are often defined as covalent adaptable networks (CANs) or vitrimers, can be reprocessed, repaired, or degraded under certain external stimuli (such as heat, pH, light, solvent, etc.) through the reversible depolymerization or exchange reactions of their dynamic cross-links [3,9,10,11]. So far, a number of CANs based on Michael addition [12,13,14], Diels-Alder reaction [15,16], disulfide exchange [17,18,19], imine metathesis [20,21,22], transesterification [23,24,25], olefin metathesis [26,27], silyl ether transalkoxylation [28,29], diketoenamine exchange [30,31], and dioxaborolane metathesis [32,33] have been proposed in the literature. Besides recyclability, many other adaptive properties of CANs were also investigated, such as reconfigurability [34,35], shape memory [36,37], and network topological transformation [18,25,38]. 

In general, reworkable thermosetting polymers contain the cleavable linkages, which can decompose under certain conditions such as specific reagents or heating [2]. Therefore, it is feasible to obtain recyclable and reworkable thermosetting polymers with multiple stimulus responsiveness by introducing dynamic and labile covalent bonds simultaneously. It was reported that polyhexahydrotriazine thermosets (PHTs) have the advantages of easy preparation, high mechanical performances, and degradability in mild acidic solutions [39,40,41]. Herein, a novel PHT was designed and prepared by a one-pot and catalyst-free polycondensation between 4-aminophenyl disulfide and paraformaldehyde (PFA). We choose to incorporate disulfide bonds into the PHT because the chemistry of disulfides is very versatile. Disulfide bonds can undergo reversible thiol–disulfide exchange and disulfide exchange by heat, UV, or catalysts [42,43,44,45]. Especially, the aromatic disulfides have the advantage of low activation energy to facilitate these exchange reactions under a moderate condition [42,46]. Disulfide bonds are also regarded as thermally cleavable linkages due to their low bond dissociation energy (210–270 kJ/mol) [47,48]. More interestingly, the incorporation of disulfide bonds can bring about the mechanochromic effect for thermosetting polymers [49]. To verify our hypothesis, the recyclability, reworkability, and mechanochromic behaviors of the prepared disulfide-containing PHT were investigated in the following study.

## 2. Materials and Methods 

### 2.1. Materials 

PFA, acetone, anhydrous ethanol, chloroform, tetrahydrofuran (THF), N-methyl-2-pyrrolidone (NMP), dimethyformamide (DMF), hydrochloric acid (HCl, 36%), and sodium hydroxide (NaOH) were purchased from Sinopharm Chemical Reagent Co., Ltd., Shanghai, China. 4-aminophenyl disulfide and 2-mercaptoethanol were purchased from Shanghai Taitan Technology Co., Ltd., Shanghai, China. All the chemicals were used as received without further purification. 

### 2.2. Preparation of PHT

**Figure 1:**
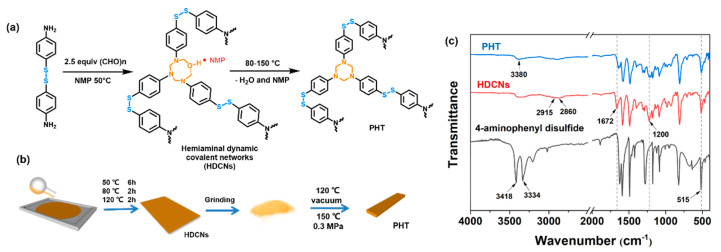
(**a**) The synthetic route of the polyhexahydrotriazine thermosets (PHT). (**b**) Preparation process of PHT bars. (**c**) FTIR spectra of hemiaminal dynamic covalent networks (HDCNs) and PHT.

Under magnetic stirring, PFA (60.0 mmol), distilled water (3.0 mL), and NMP (27.0 mL) were added into a flask and heated at 80 °C for 0.5 h. When the obtained solution was cooled down to 50 °C, 4-aminophenyl disulfide (24 mmol) was introduced and stirred for 1 h. Then, the prepolymer solution was poured into a polytetrafluoroethylene mold lined with a layer of aluminum foil, which was followed by removing the solvent and staged precuring at 50 °C for 6 h, 80 °C for 2 h, and 120 °C for 2 h. After that, the B-stage polymer film was peeled from the aluminum foil and grinded into fine powders. The obtained powders were heated at 120 °C for 4 h under reduced pressure to remove excess NMP. In order to prepare the PHT specimens with no bubbles (from evaporation of the residue NMP solvent), the dry powders were placed into the steel punch molds with specific dimensions and pressed at 150 °C under 0.3 MPa for 1 h. After cooling to room temperature, the obtained PHT specimens were polished for thermal and mechanical tests. The above preparation procedures for the PHT are illustrated in Figure 1b. 

### 2.3. Reprocessing of PHT

The cured PHT was first grinded into fine powders, and placed into a steel punch mold. Then, the PHT powders was pressed at 150 °C under 0.3 MPa for 1 h. After cooling and demoulding, defect-free PHT specimens were obtained. This reprocessing treatment was repeated twice.

### 2.4. Chemical Reworkability

Chemical degradation of the PHT was performed in acid and thiol solutions, respectively. Typically, a piece of PHT film (about 300 mg) was placed into 15 mL of HCl (1 M) or 15 mL 2-mercaptoethanol/DMF (0.1 M) solutions, which was followed by standing for 24 h.

### 2.5. Thermal Reworkability

The prepolymer solution in Section 2.2 was poured onto a glass slide and heated at 50 °C for 6 h, 80 °C for 2 h, 120 °C for 2 h, and 150 °C for 1 h. The obtained PHT cured on the glass slide was thermally degraded at 200 °C for 1 h in an oven. The degradation products were removed from the glass slide by a clean cloth soaked by acetone.

### 2.6. Characterizations

Fourier transform infrared spectroscopy (FTIR) was carried out on a Nicolet 6700 FTIR spectrometer using the attenuated total reflection (ATR) mode with a scan range from 400 to 4000 cm^-1^. Dynamic mechanical analysis (DMA) was performed on a TA Instruments DMA Q800 with a heating rate of 3 °C min^−1^ and a frequency of 1 Hz. Double cantilever mode was used for the DMA tests and the specimen dimension was 60 × 15.0 × 2.0 mm^3^. Stress-relaxation tests were also performed on a TA Instruments DMA Q800. The tests were carried out using the tensile mode with rectangular specimens (12.0 × 6.0 × 1.0 mm^3^). The specimen was aligned by preloading 10^-3^ N force and thermally equilibrated for 10 min at each test temperature. The data of relaxation modulus versus time were recorded. Flexural tests, which followed the ASTM D790-03 standard, were performed on the Wance ETM104B-EX electronic universal testing machine with a 2000 N load cell at a crosshead speed of 1 mm min^−1^. Each reported value was the average of at least three valid specimens. Thermal gravimetric analysis (TGA) was conducted on a TA Instruments TGA Q 500 using nitrogen as the purge gas at a heating rate of 10 °C min^-1^ from 50 to 800 °C.

## 3. Results

### 3.1. Preparation of PHT

First, hemiaminal dynamic covalent networks (HDCNs) were synthesized by a water-catalyzed stepwise condensation of 4-aminophenyl disulfide with PFA at 50 °C [39]. The HDCNs were further cyclized at high temperature to yield the PHT (Figure 1a). Similar to the reported method by Yuan et al. [41], the specimens for thermal and mechanical tests were prepared as illustrated in Figure 1b. Since both the disulfide bond and hemiaminal group are dynamic covalent bonds [50,51], uniform coherent PHT bars could be obtained by pressing HDCN powders at 150 °C under 0.3 MPa for 1 h. It should be noted that the PHT specimens were prepared by a hot-press process rather than direct curing in a mold. This is because there exists a high boiling point NMP solvent in the monomer mixture. If the mixture is directly cured in a mold, this would generate bubbles in the PHT specimens owing to evaporation of the residue NMP solvent. Considering that the introduced dynamic disulfide bonds can endow the PHT with re-processability [50]. A hot-press process was used to prepare the PHT specimens with high quality. The PHT formation was characterized by FTIR. Figure 1c shows the FTIR spectrum of HDCNs after precuring. Compared with the FTIR spectrum of 4-aminophenyl disulfide, the new band at 1200 cm^−1^ is assigned to the C-N stretching vibration in the hemiaminal group. The bands at 2860 and 2915 cm^−1^ can be attributed to asymmetric and symmetry stretching vibration from the -CH2- on the hexatomic ring. The characteristic bands of primary amine (3300 and 3190 cm^−1^) in 4-aminophenyl disulfide no longer exist, and a broad band between 3200–3400 cm^−1^ attributed to -O-H stretching vibration [52] appears in the FTIR spectrum of HDCNs. The band at 1672 cm^-1^ can be assigned to the C=O stretching vibration from the residual NMP solvent in HDCNs [41]. The above results indicate that HDCNs are not fully cyclized at 120 °C. After vacuum drying (120 °C) and hot pressing at 150 °C (0.3 MPa) for 1 h, the band at 1672 cm^−1^ corresponding to the C=O stretching vibration from the NMP solvent almost disappears in the FTIR spectrum of PHT (Figure 1c) [52]. It should be noted that the unexpected weak band at 3380 cm^−1^ attributed to the -O-H stretching vibration may be due to the side reactions reported in the literature [41,53]. In addition, the band at 515 cm^−1^ assigned to the S-S stretching vibration is retained during the preparation of the PHT.

### 3.2. Reprocessing and Healing of PHT

**Figure 2:**
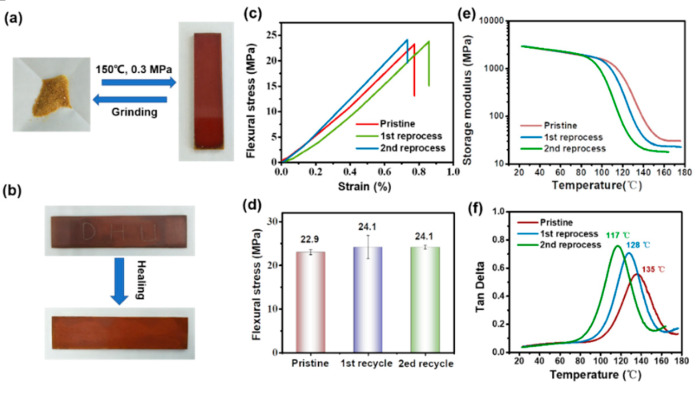
(**a**) Reversible reprocessing of the PHT. (**b**) A scratched PHT bar before and after healing. (**c**) Representative flexural stress versus strain curves (**d**) and flexural stress of pristine and reprocessed PHTs. (**e**) Storage modulus and (**f**) tan δ versus temperature curves of pristine and reprocessed PHTs.

The reported hexahydrotriazine-containing thermosets are degradable in acid solutions, but they cannot be reprocessed or healed [39,40,41,54]. Herein, the incorporated disulfide bonds, which can undergo bond exchange reactions at elevated temperature, endow the PHT with vitrimer features [50,55,56]. The PHT powders are able to be reprocessed into new coherent bulk specimens by hot pressing at 150 °C under 0.3 MPa for 1 h (Figure 2a). This process can be repeated several times. The PHT is also healable, as shown in Figure 2b. A PHT bar with scratches can be easily healed by an electric iron or hot-press treatment using the above temperature. The mechanical properties of pristine and reprocessed PHTs were investigated by three-point bending tests. Figure 2c,d shows the representative flexural stress versus stain curves and flexural stress of pristine and reprocessed PHTs after different reprocessing cycles. As can be seen, the PHT exhibits good re-processability and two generations of reprocessed PHTs even show slightly increased average flexural stress when compared to the pristine PHT.

The thermomechanical properties of pristine and reprocessed PHTs were studied by dynamic mechanical analysis (DMA). The storage modulus and tan δ versus temperature curves are shown in Figure 2e,f. Storage modulus can reflect the stiffness of polymer materials. The pristine and two reprocessed PHTs exhibit similar storage moduli (~2890–2910 MPa) at room temperature, which are comparable to those of conventional thermosetting polymers such as epoxy resins. The temperature at the maximum tan δ is regarded as the glass-transition temperature (*T*_g_). It is shown that the *T*_g_s of reprocessed PHTs continuously decrease with the reprocessing cycles. Compared with the pristine PHT, the *T*_g_ of the second reprocessed PHT decreases from 135 to 117 °C. This might be because the disulfide bond, as a type of weak covalent bond, could be cleaved by oxidation during the hot-press treatment [2], leading to the decreased crosslinking density of reprocessed PHTs.

### 3.3. Thermal Stress Relaxation

**Figure 3:**
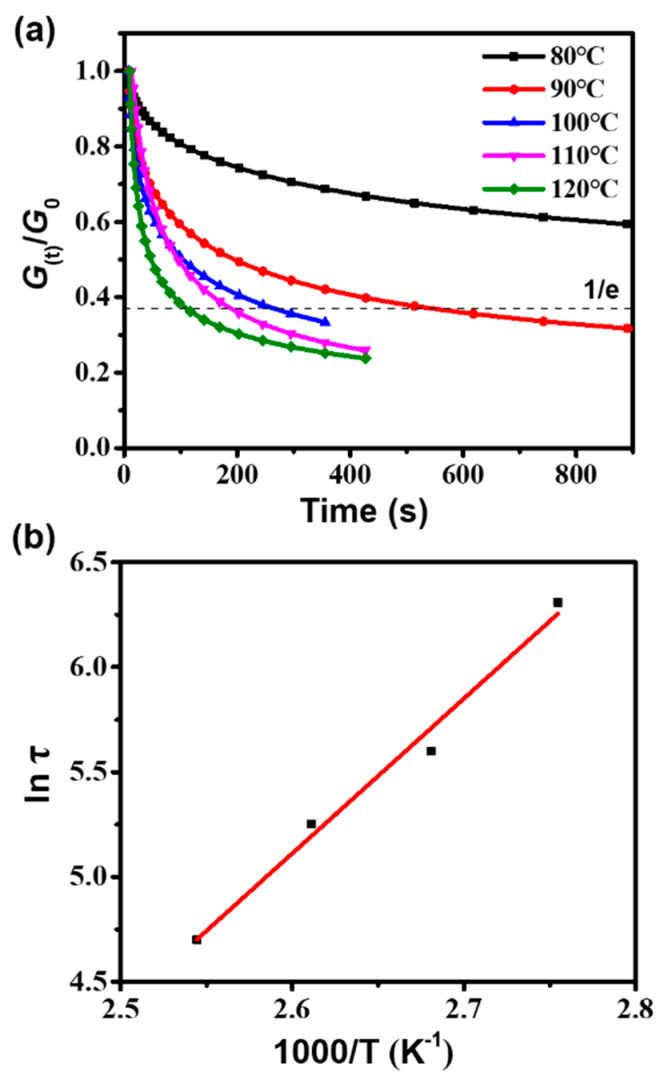
(**a**) Normalized relaxation modulus as a function of time of the polyhexahydrotriazine thermoset (PHT) at different temperatures. (**b**) The Arrhenius fitted line between ln (*τ*) and 1000/T.

As a CAN (or vitrimer), the dynamic structural characteristics of the PHT were studied by thermal stress relaxation tests. Figure 3a shows the normalized relaxation modulus *G*_(t)_/*G*_0_ of the PHT as a function of time at different temperatures. The PHT is able to relax the stress quickly when heated to more than 90 °C. The curves of stress relaxation are observed to cross at 100 and 110 °C. This is because these two temperatures are both close to the onset region of the glass transition and the temperature difference is small. The PHT shows similar quick stress relaxation behaviors in the initial stage of the tests at 100 and 110 °C. A slight fluctuation of the DMA measurement might cause the cross of stress relaxation curves. Similar phenomena were also found for the reported vitrimers [50,55,57]. Following Maxwell’s viscoelastic fluid model, the relaxation time (τ) is defined as the time when the sample is relaxed to 1/e of the initial modulus [8]. As can be seen, the stress relaxation of the PHT at 80 °C is slow, and the τ value at 80 °C is estimated to be more than 2000 s by extrapolating the curve of the relaxation modulus versus time (Figure 3a). As expected, the *τ* values decrease with the elevation of temperature, owing to the increased exchange rate of disulfide bonds. The effect of temperature on the stress relaxation will be discussed at the end of this section. The activation energy (*E*_a_) of disulfide bond exchange reactions can be calculated via Arrhenius’ law [50,58], as shown in Equation (1).

(1) $$\tau \left(T\right)=\tau_{0}\;exp\left(\frac{E_{a}}{RT}\right)$$

where *τ* is the relaxation time, *τ*_0_ is the characteristic relaxation time at infinite temperature, *T* is the testing temperature, and *R* is the universal gas constant. The Arrhenius relationship of ln (*τ*) versus 1000/*T* (*T* is the testing temperature) is shown in Figure 3b. The calculated *E*_a_ of disulfide bond exchange reactions is 61 kJ mol^-1^, which is comparable to that (55 kJ mol^-1^) of the disulfide-containing epoxy resin [50]. The *E*_a_ of the PHT is lower than many reported vitrimers containing other dynamic covalent bonds, such as ester, imine, carbamate, and triazolium [8,21,59,60,61].

Since the exchange reactions between disulfide bonds are temperature-dependent behaviors, the topology freezing transition temperature (*T*_v_) is another important characteristic parameter for the PHT. *T*_v_ is a hypothetical temperature at which vitrimers convert from solid to liquid and have a viscosity of 10^12^ Pa.s [50,62]. It is generally accepted that the crosslinking networks of vitrimers would be frozen when the temperature is below *T*_v_, owing to the low exchange reaction rate. The relaxation time (*τ*) at *T*_v_ can be calculated using Equation (2) [57].

(2) $$\eta =\frac{1}{3}E^{\prime }\tau$$

where *E*’ is the plateau modulus in the rubber region from the DMA curve (Figure 2e) and *η* is the viscosity at *T_v_* (i.e., 1012 Pa.s). The calculated *τ* (at *T_v_*) was applied to the Arrhenius’ fitted line (Figure 3b) to obtain *T_v_*. The *T_v_* of the PHT is −85.7 ℃, which is consistent with the fact that the exchange reactions between aromatic disulfides can occur at room temperature [42]. It is noteworthy that the disulfide exchange reactions in the PHT networks are also determined by the segmental mobility related to *T_g_*. In general, it is believed that large-scale coordinated motions of the polymer chains occur in the glass transition region. When the PHT is in the glassy state, the disulfide bonds in the PHT would have very low contact probability. However, a slow stress relaxation of the PHT is observed at 80 °C, which is below its *T_g_* (135 ℃), as shown in Figure 3a. Similar stress relaxation below the *T_g_* of the vitrimer was also reported by Zhao and his co-workers [63]. This indicates the bond exchange reactions between the dynamic covalent bonds in the vitrimer could occur and cause stress relaxation even if the motion of polymer chains is slow. The polymer chains of the PHT should already have certain mobility at 80 °C to facilitate the disulfide bond exchange reactions. Considering that the *T_g_* (135 °C) of the PHT is much higher than its *T_v_*, the stress relaxation phenomenon is dominated by *T_g_* and, thus, gradually becomes pronounced as the temperature approaches *T_g_.*

### 3.4. Reworkability and Solvent Resistance

**Figure 4:**
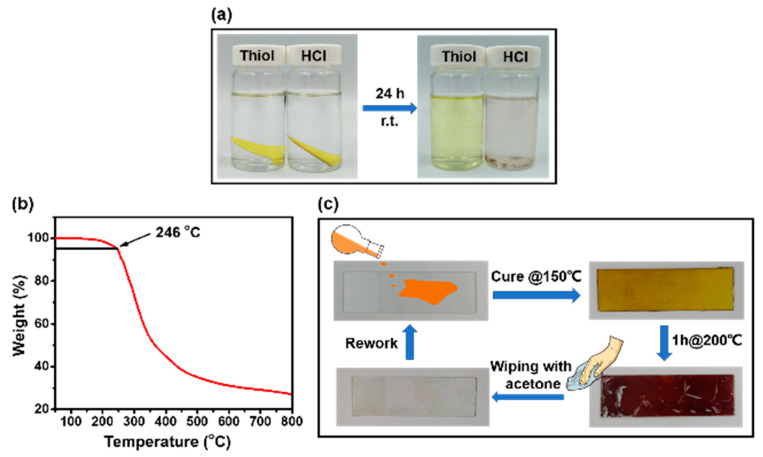
(**a**) Chemical degradation of the PHT in 2-mercaptoethanol/DMF and HCl (1M) solutions. (**b**) TGA curve of the PHT under a nitrogen atmosphere. (**c**) Thermal re-workability of the PHT at 200 °C for 1 h.

Reworkable thermosetting polymers mean they can be thoroughly removed by structure break-down under controlled external stimulus, such as chemical or heat treatments [2,4,64]. To endow thermosetting polymers with re-workability, a well-established strategy is to introduce chemically- or thermally-labile groups [2]. It has been reported that the hexahydrotriazine ring tends to degrade in the aqueous acid solution [41,54], and the disulfide bond can react with thiols through the bond exchange reaction [50,65]. To examine the chemical reworkability of the PHT, two pieces of PHT samples were immersed in 2-mercaptoethanol/DMF (0.1M) and HCl (1M) solutions, respectively. As shown in Figure 4a, the PHT could be entirely dissolved in the thiol solution, which turned pale yellow after standing for 24 h at room temperature. The PHT was disintegrated into powders and partially dissolved the HCl solution at the same condition. The PHT is more liable to degrade in the thiol solution, which is consistent with the low *E_a_* of disulfide bond exchange reactions.

It has been reported that thermally reworkable thermosetting polymers for electronic packaging applications are desirable to decompose at a temperature around 220 °C [66]. The thermal stability of the PHT was investigated by TGA (Figure 4b). The PHT starts to decompose at 246 °C (5% weight loss) at a heat rate of 10 ℃ min^-1^. To demonstrate its thermal reworkability, the PHT was cured on a glass slide. After heating at 200 ℃ for 1 h, the PHT decomposed and the dark brown residue on the glass slide could be conveniently wiped off using a piece of cloth soaked by acetone (Figure 4c). The above demonstrations suggest that the PHT can be reworked by both chemical and thermal methods. 

**Figure 5:**
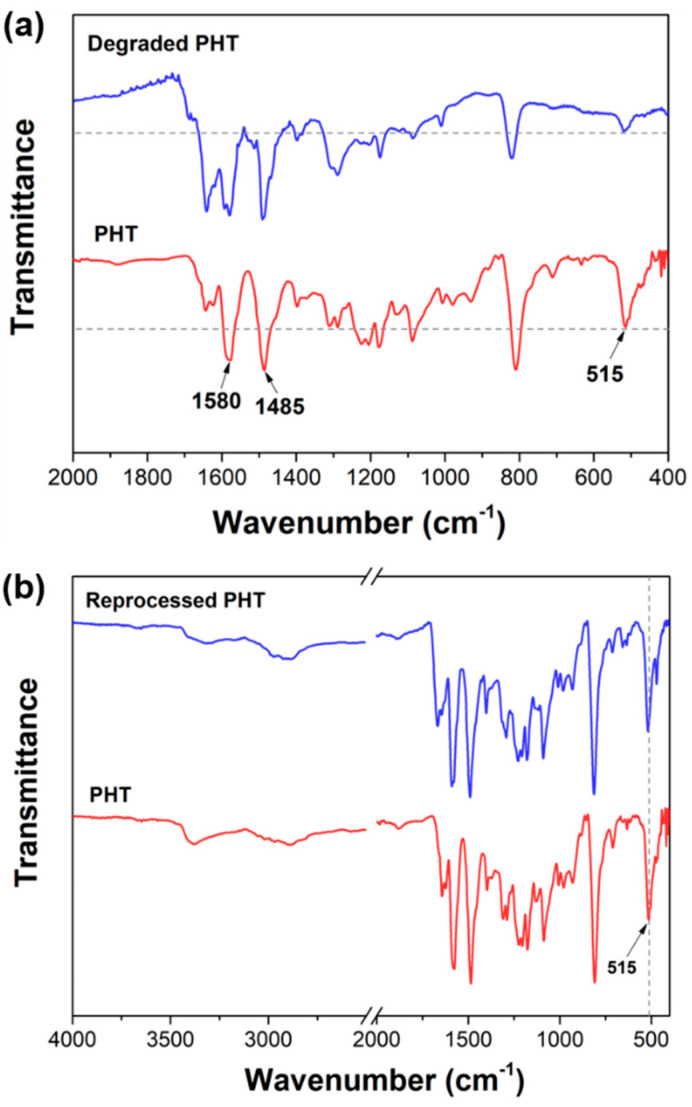
(**a**) FTIR spectra of the PHT and PHT residue after heating at 200 °C. (**b**) FTIR spectra of the PHT and reprocessed PHT after the hot-press treatment at 150 °C.

The thermal degradation of the PHT at 200 °C was studied by analysing the structure of the residue by FTIR (Figure 5a). The bands attributed to the stretching vibration of benzene ring (1580 and 1485 cm^−1^) are used as internal references. Compared with the pristine PHT, the band intensity of S-S stretching vibration (515 cm^−1^) clearly decreases in the FTIR spectrum of the PHT residue, indicating the cleavage of disulfide bonds. This is consistent with the lower low bond dissociation energy (210–270 kJ/mol) of disulfide bonds [47,48]. In addition, it has been reported that there might exist some weak chemical bonds in the PHT (such as tertiary amine-containing ether linkages), which could also make the PHT start to degrade at around 200 °C [41]. Considering the lower degradation temperature of the PHT, it is essential to verify the structure of the reprocessed PHT after the hot-press treatment (150 °C, 0.3 MPa) adopted in this work. Compared with the pristine PHT, no significant changes are observed in the FTIR spectrum of the reprocessed PHT, and the band (515 cm^-1^) attributed to disulfide bonds is well maintained (Figure 5b). This indicates that disulfide bonds are not cleaved during the hot-press treatment. It is acceptable to reprocess the PHT at 150 °C based on the disulfide exchange reactions.

**Figure 6:**
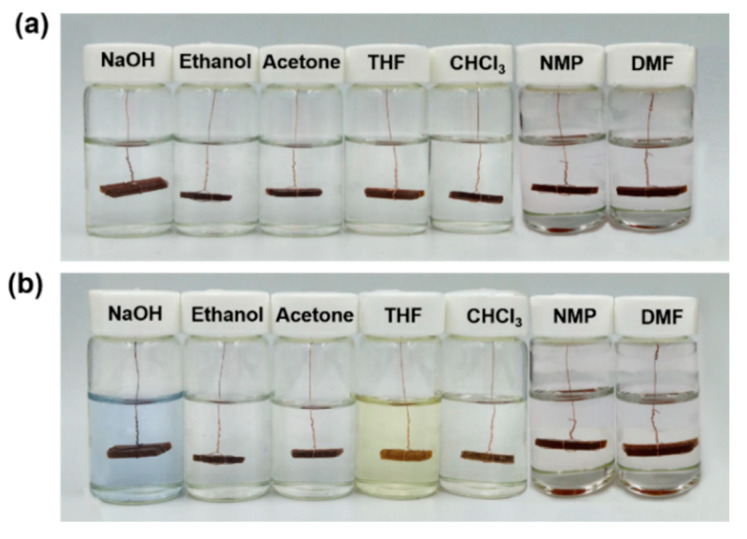
Photographs of the PHT in different solvents (**a**) before and (**b**) after standing for 120 h at room temperature.

Owing to the chemical sensitivities of the hexahydrotriazine ring and disulfide bond, it is essential to evaluate the solvent resistance of the PHT. Figure 6 shows the photographs of PHT specimens immersed in NaOH (1M), ethanol, acetone, THF, chloroform, NMP, and DMF for 120 h at room temperature. As can be observed, the PHT exhibits superior solvent resistance in the aqueous alkali solution and organic solvents except that a fraction is dissolved THF. This confirms that the degradation of the PHT has specific stimulus responsiveness. Like conventional thermosetting polymers, PHT is a suitable material for a range of applications.

### 3.5. Mechanochromism

**Figure 7:**
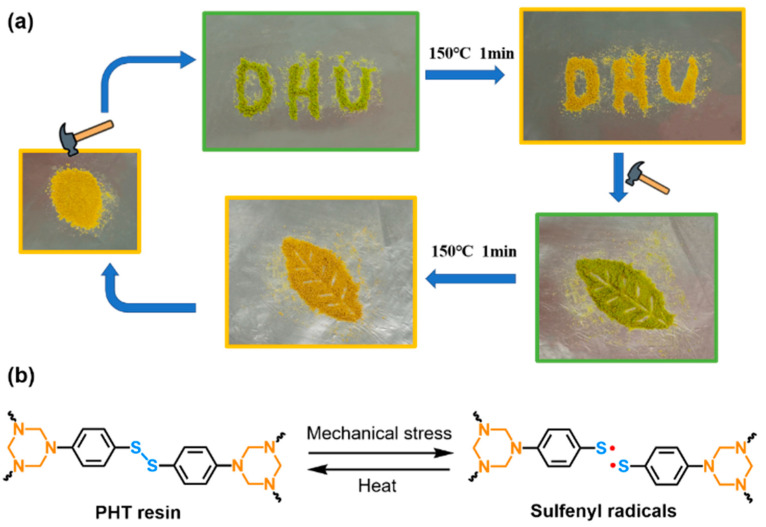
(**a**) Reversible mechanochromic behaviors of the PHT. (**b**) A schematic illustration of the generation and coupling of sulfenyl radicals in the PHT networks.

Interestingly, the PHT shows reversible mechanochromic behaviors, as shown in Figure 7a. The PHT powders could transiently turn from golden yellow to green when hit using a hammer. The colour of the PHT would totally recover after heating at 150 °C only for 1 min. When the PHT resin was hit again, its colour turned green accordingly. It is worth noting that only breaking is able to induce this mechanochromic effect, while stretching or bending the PHT will not cause any colour changes. It was proposed that the green coloration was related to the formation of sulfenyl radicals generated from the mechanical cleavage of disulfide bonds (Figure 7b) [49]. Although these sulfenyl radicals are quite reactive, they can be “frozen” and survive for a certain time when the temperature is far below the *T*_g_. As the segmental mobility increases at high temperature, these sulfenyl radicals tend to pair up to disulfide bonds again, resulting in the colour recovery. Therefore, the PHT has the inherent self-monitoring function for mechanical damages.

## 4. Conclusions

In summary, we report a disulfide-containing PHT with a combination of recyclability, re-workability, and damage monitoring function. The PHT can be reprocessed like thermoplastics by hot pressing. Notably, the reprocessed PHTs exhibit almost a full recovery of flexural strength even after two reprocessing cycles. The results of stress relaxation tests indicate that the PHT has lower bond exchange activation energy (61 kJ mol^−1^) and topology freezing transition temperature (−85.7 ℃) due to the dynamic feature of disulfide bonds. The PHT can be reworked by both chemical and thermal manners. The PHT is liable to degrade in the mild aqueous acid and thiol solutions at room temperature, but shows good resistance to many conventional organic solvents. The removal test demonstrates that the residuals from the decomposed PHT (200 °C for 1 h) can be easily removed by acetone. Finally, the PHT shows reversible mechanochromic behaviors due to the generation and coupling of sulfenyl radicals. It is foreseen that the simple preparation and multiple functions make the PHT have great potential for a variety of applications, such as electronic encapsulation and fiber-reinforced polymer composites.
